# Low transmission of *Wuchereria bancrofti* in cross-border districts of Côte d’Ivoire: A great step towards lymphatic filariasis elimination in West Africa

**DOI:** 10.1371/journal.pone.0231541

**Published:** 2020-04-13

**Authors:** Firmain N. Yokoly, Julien B. Z. Zahouli, Aboulaye Méite, Millicent Opoku, Bernard L. Kouassi, Dziedzom K. de Souza, Moses Bockarie, Benjamin G. Koudou

**Affiliations:** 1 Unité de Formation et de Recherche Sciences de la Nature, Université Nangui Abrogoua, Abidjan, Côte d’Ivoire; 2 Centre Suisse de Recherches Scientifiques en Côte d’Ivoire, Abidjan, Côte d’Ivoire; 3 Centre d’Entomologie Médicale et Vétérinaire, Université Alassane Ouattara, Bouaké, Côte d’Ivoire; 4 Programme National de Lutte contre les Maladies Tropicales Négligées à Chimiothérapie Préventive, Ministère de la Santé, Abidjan, Côte d’Ivoire; 5 Department of Parasitology, Noguchi Memorial Institute for Medical Research, University of Ghana, Legon, Accra, Ghana; 6 European & Developing Countries Clinical Trials Partnership, Cape Town, South Africa; 7 Department of Medicine, University of Cape Town, Cape Town, South Africa; University of California Berkeley, UNITED STATES

## Abstract

**Background:**

Lymphatic filariasis (LF) is widely endemic in Côte d’Ivoire, and elimination as public health problem (EPHP) is based on annual mass drug administration (MDA) using ivermectin and albendazole. To guide EPHP efforts, we evaluated *Wuchereria bancrofti* infection indices among humans, and mosquito vectors after four rounds of MDA in four cross-border health districts of Côte d’Ivoire.

**Methodology:**

We monitored people and mosquitoes for *W*. *bancrofti* infections in the cross-border health districts of Aboisso, Bloléquin, Odienné and Ouangolodougou, Côte d’Ivoire. *W*. *bancrofti* circulating filarial antigen (CFA) was identified using filariasis test strips, and antigen-positive individuals were screened for microfilaremia. Moreover, filarial mosquito vectors were sampled using window exit traps and pyrethrum sprays, and identified morphologically at species level. *Anopheles gambiae* s.l. and *Culex quinquefasciatus* females were analyzed for *W*. *bancrofti* infection using polymerase chain reaction (PCR) technique.

**Principal findings:**

Overall, we found a substantial decline in *W*. *bancrofti* infection indices after four rounds of MDA compared to pre-MDA baseline data. CFA prevalence fell from 3.38–5.50% during pre-MDA to 0.00–1.53% after MDA interventions. No subjects had detectable levels of CFA in Ouangolodougou. Moreover, post-MDA CFA prevalence was very low, and below the 1% elimination threshold in Aboisso (0.19%) and Odienné (0.49%). Conversely, CFA prevalence remained above 1% in Bloléquin (1.53%). *W*. *bancrofti* microfilariae (Mf) were not found in Aboisso, Bloléquin, and Ouangolodougou, except for Odienné with low prevalence (0.16%; n = 613) and microfilaremia of 32.0 Mf/mL. No *An*. *gambiae* s.l. and *Cx*. *quinquefasciatus* pools were infected with *W*. *bancrofti* in Bloléquin and Ouangolodougou, while they exhibited low infection rates in Aboisso (1% and 0.07%), and Odienné (0.08% and 0.08%), respectively.

**Conclusions:**

In cross-border areas of Côte d’Ivoire, LF infection indices in humans and mosquito vectors substantially declined after four rounds of MDA. CFA prevalence fell under the World Health Organization (WHO)-established threshold (1%) in Aboisso, Ouangolodougou and Odienné. Moreover, *W*. *bancrofti* prevalence in mosquitoes was lower than WHO-established threshold (2%) in all areas. This might suggest the interruption of *W*. *bancrofti* transmission, and possible MDA cessation. However, a formal transmission assessment survey (TAS) and molecular xenomonitoring in mosquito vectors should be implemented before eventual MDA cessation. However, MDA should pursue in Bloléquin where *W*. *bancrofti* infection prevalence remained above 1%. Our results provided important ramifications for LF control efforts towards EPHP in Côte d’Ivoire.

## Introduction

Lymphatic filariasis (LF) is a neglected tropical disease that continues to be a major cause of morbidity and permanent disability in endemic populations [1, 2]. In 2018, 893 million people across 49 countries were living in at risk of LF and required preventive chemotherapy to stop the spread of infection [3]. Infection is caused by a mosquito-transmitted filarial worm and, if left untreated, can lead to permanent and debilitating disability.

As of 1997, LF was endemic in 73 tropical and sub-tropical countries where parasites had already infected over 120 million people, with 40 million people suffering from complications [4, 5]. In recognition of the significant worldwide burden of LF, the World Health Organization (WHO) launched the Global Program to Eliminate Lymphatic Filariasis (GPELF) in 2000 to achieve disease elimination as public health problem (EPHP) by 2020. For interruption of transmission, the strategy is annual single dose mass drug administration (MDA) of albendazole in combination with diethylcarbamazine or ivermectin to the LF endemic communities [5, 6]. In 2011, the WHO published guidelines for halting treatment and verifying EPHP through the use of transmission assessment surveys (TAS) to measure a target threshold; 1% of microfilariae (Mf) prevalence [7]. By October 2018, 51 of the 72 LF endemic countries have fully implemented MDA [3, 8]. WHO acknowledged after post-MDA validation in 16 countries and territories (including Cambodia, Cook Islands, Egypt, Kiribati, Maldives, Marshall Islands, Niue, Palau, Sri Lanka, Thailand, Togo, Tonga, Vanuatu, Viet Nam, Wallis and Fortuna, and Yemen [3, 8]. Thus, LF is no longer a public health problem in these countries and territories, and 597 million people no longer require preventive chemotherapy [1]. As more countries progress towards EPHP, it is crucial that this process is well-informed, as prematurely halting treatment and surveillance programs could pose a serious threat to global progress [2].

In the African region, where up to 464 million people in 33 countries live in endemic areas [9]. In sub-Saharan Africa, LF is caused by infection with the parasitic nematode *Wuchereria bancrofti* transmitted by *Anopheles* and *Culex* mosquitoes [10]. The main filarial vectors are *Anopheles gambiae* and *An*. *funestus* group in rural, and *Culex quinquefasciatus* in urban and semi-urban areas [11, 12]. In West Africa, *An*. *gambiae* and *Cx*. *quinquefasciatus* are known as the primary vectors of *W*. *bancrofti* [13–15]. Togo (West Africa) became the first country in sub-Saharan Africa to receive WHO validation of EPHP [4], while others like Benin and Ghana are advanced at transmission assessment survey (TAS) phase [16]. Moreover, border countries of Côte d’Ivoire (West Africa) including Burkina Faso, Ghana, Guinea, Liberia and Mali are endemic for LF, have implemented MDA since several years, and have made significant progress towards EPHP achievement [17, 18].

Côte d'Ivoire is broadly endemic for LF [2, 17]. According to Brengues et al. [19], *An*. *gambiae* is the major vector of LF in Côte d’Ivoire. More than 83% of its populations are estimated to be at *W*. *bancrofti* infection risk areas. Out of the 112 health districts, 98 are FL endemic and eligible for MDA. After pre-MDA assessment in 2014, the National Program for Neglected Tropical Diseases Control through Preventive Chemotherapy (NPNTDCPC) of Côte d’Ivoire embarked on its first MDA intervention based on a combination of ivermectin and albendazole. MDA interventions started only October 2014 due to financial support and logistical challenges related to socio-political unrest. The overall national therapeutic coverage is estimated at 74.2% in 2018 [20]. The initial pre-MDA prevalence of FL in cross-border Ivorian health districts, such as Aboisso, Bloléquin, Odienné and Ouangolodougou varied between 3.38% and 5.50% (> 1%), and were thus eligible for MDA [2–5]. These districts then received the first MDA in October 2014, and last (sixth) round in April 2019. Importantly, these health districts share borders with the health districts of neighboring countries that have already received several rounds of MDA or stopped MDA activities or move to pre-TAS or TAS [2, 17]. In such a particular context, monitoring the impact of MDA intervention is crucial for measuring the success of the LF elimination programmes, and to prevent the rebound of disease on both sides of the borders. In the present parasitological and entomological study was conducted between July 2016 and December 2017. We assessed *W*. *bancrofti* infection indices in human populations, and mosquito vectors (*An*. *gambiae* s.l. and *Cx quinquefaciatus*) in four cross-border health districts of in Côte d’Ivoire after four rounds of MDA to guide LF elimination efforts. We hypothesized that *W*. *bancrofti* infections indices are low in theses cross-border health districts after the four rounds of MDA interventions. The key results provide valuable information and recommendations for decision making, including a possible cessation of MDA, and additional actions towards LF EPHP achievement in Côte d’Ivoire.

## Methods

### Ethics statement

The surveys were conducted in accordance with the study protocol approved by the Institutional Ethics Review Board of the Liverpool School of Tropical Medicine (1189RS) and from the Comité Nationale d’Ethique des Sciences de la Vie et de la Santé (CNESVS) from the Republic of Côte d’Ivoire (001//MSHP/CNER-kp) Written informed consent was obtained from individuals aged 18 years and above. For minors (aged <18 years), written informed consent was obtained from parents or legal guardians, while minors provided oral assent. In some households, oral rather than written informed consent was obtained due to illiteracy. CNESVS explicitly approved our consent procedures. Participants were informed about the purpose and procedures of the study, including potential risks and benefits. The data were analysed and reported to exclude any directly identifiable information, in order to maintain anonymity of participants.

### Study area

**Fig 1:**
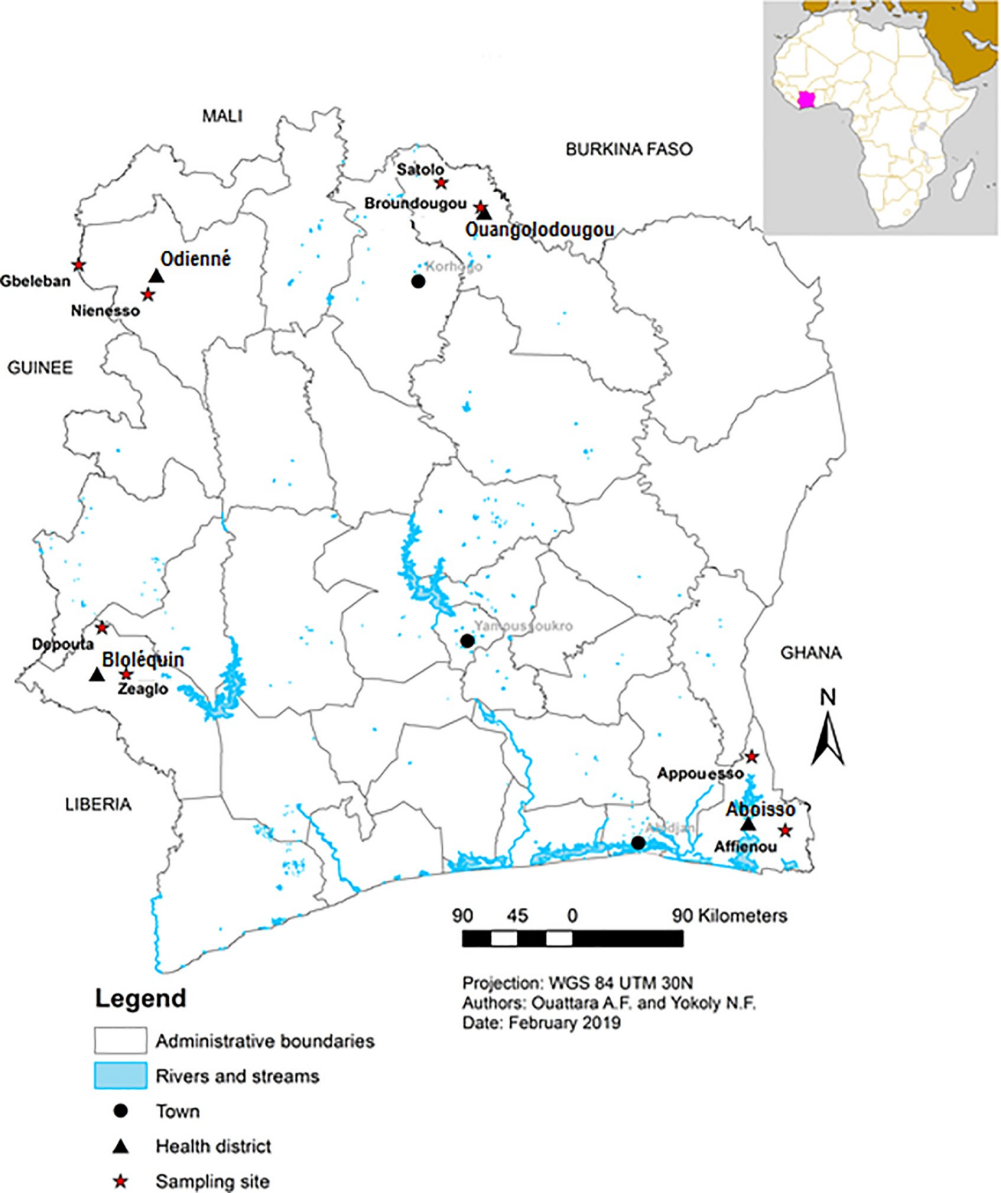
Location of the study sites in cross-border health districts of Côte d’Ivoire. This map was created using Arcmap version 10.

The study was conducted in four cross-border health districts, namely Aboisso, Bloléquin, Odienné and Ouangolodougou across different regions of Côte d’Ivoire (Fig 1).

The district of Aboisso (5° 28’ N, 3° 12’ W) is located in southeastern Côte d’Ivoire at southwestern border of Ghana. The climate is humid tropical type characterized by abundant rainfall with an average annual height of about 1,500 mm of rain. The average annual temperatures are between 25 and 27°C. It expands over 4,662 km^2^ with a population size of 307,852 people, thus a density of 66 inhabitants per km^2^. Coffee, cocoa, rubber and palm oil are the main cash crops while vegetable, taro and banana are the main food crops in the area.

The district of Bloléquin (6° 34’ N, 8° 00’ W) is situated in the forest zone of west of Côte d’Ivoire at the border of Liberia. The population is estimated at 123,336 inhabitants. The climate is mountainous type with annual average rainfall sometimes exceeding 2,000 mm per year and annual temperatures ranging from 15 to 33°C. It covers an area of 2,962 km^2^ with a population density of approximately 41 inhabitants per km^2^. Coffee, cocoa and rubber are the main agricultural activity of this region. Food crops are dominated by banana, cassava, maize, rice and vegetables

The district of Odienné (9° 30’ N, 7° 33’ W) is located in savannah zone in north-west of Côte d’Ivoire and bordered in its western part by the Republic of Guinea. It covers an area of 14 000 km^2^ with a population of 193,364, giving a density 13.8 inhabitants per km^2^. The climate is tropical sub-humid type with the annual rainfall amounts vary between 1,400 and 1,600 mm per year and the average annual temperatures ranged between 25.4 and 33°C. The most dominant agricultural products in this setting are cereals, tubers, cotton and cashew nuts

The district of Ouangolodougou (9° 58' N, 5° 09' W) is located in the savannah zone at the north of Côte d’Ivoire. It bordered in the northern part by Burkina Faso and covers an area of 5,380 km^2^, with an estimated population of 260,519 habitants, hence, a density of 48.4 inhabitants per km^2^. The climate is Sudanese type with the annual rainfall varies between 1,000 mm and 1,400 mm and annual temperatures range from 15 to 34°C. The main agricultural activities are cotton, cashew nut, onion, groundnut and food crops (e.g., cereals, rice, and yams). The residencies of the collection areas in all districts are composed of traditional and modern houses.

Since October 2014, all the four cross-border health districts received annually MDA based on ivermectin and albendazole until December 2017 (i.e., end date of our study), and December 2019. The therapeutic coverage in these health districts from 2014 to December 2017 (i.e., end date of our study) varied between 65.6 and 76.6% (S1 Table).

### Study design

The parasitological and entomological surveys were conducted in four cross-border health districts of Côte d’Ivoire, namely Aboisso, Bloléquin, Odienné and Ouangolodougou. Each health district was represented by two sentinel sites; Affienou and Appouesso in Aboisso, Zeaglo and Depouta in Bloléquin, Gbeleban and Nienesso in Odienné, and Broundougou and Satolo in Ouangolodougou. We conducted filariasis test strip (FTS) in December 2017 all the eight sentinel site after the fourth MDA round. The collection of adult filarial mosquito vectors was performed monthly in all sentinel sites. The first phase of entomological collections (July-December 2016) were conducted after three MDA rounds (2014, 2015, and 2016). The second phase of entomological collections (July-December 2017), and parasitological surveys (December 2017) were carried out after four MDA rounds (2014, 2015, 2016, and 2017). FTS was performed after (three weeks) the fourth MDA rounds.

We did not carry out any specific pre-MDA surveys for this study. We obtained the pre-MDA baseline data on FTS surveys from NPNTDCPC, with different study design (e.g., number of community members involved) compared to post-MDA. Moreover, *W*. *bancrofti* microfilaremia data for the pre-MDA period were not available. The pre-MDA baseline entomological data were not available as well. Thus, entomological data derived from the post-MDA mosquito collections only.

### Detection of *W*. *bancrofti* antigen in blood

We employed FTS (Alere Scarborough, Maine, USA) method as described in Weil et al. [21], for the detection of circulating filarial antigen in finger-prick blood samples taken during the day. We conducted FTS with a purpose to verify the absence of LF transmission to people. Only male and female individuals’ aged ≥ 5 years from the four districts were included in the survey. In total, we selected and surveyed 2,409 (1,221 males and 1,188 females), including 526 individuals (251 males and 275 females) in Aboisso, 721 individuals (369 males and 352 females) in Bloléquin, 613 individuals (334 males and 279 females) in Odienné, and 549 individuals (267 males and 282 females) Ouangolodougou. The FTS devices were stored at room temperature and carried to the field in cooled polystyrene foam boxes. In each community, blood from each eligible participant was tested directly in the field by FTS according to the manufacturer’s instructions. Briefly, 75μL of finger-prick blood collected was applied to the sample application pad of the FTS. All results were read strictly after 10 min. Subjects with positive FTS results were followed up for night blood collections to screen for microfilariae.

### Sampling of mosquitoes

We collected adult mosquito samples using window exit traps (ETC) and pyrethrum knock-down spray sheet collections (PSC). In each sentinel site, 15 exit traps were installed on the windows of the households. ETC collections were performed monthly using hemolysis tubes for two consecutive days in each district between 6 a.m. to 9 a.m. to sample exophilic mosquitoes. PSC sampling was conducted monthly within bedrooms between 6 a.m. and 9 a.m., and consisted of capturing resting mosquitoes inside houses. In each sentinel site, 20 households were longitudinally sprayed. PSC were performed in households different from those benefiting from the ETC collections. However, in case of unavailability or refusal of participants, mosquitoes were collected from neighboring households.

### Mosquito species identification and dissection

Mosquitoes collected in ETC and by PSC were identified to the species level on the basis of morphological criteria using readily available identification keys [22, 23]. After determining their feeding status, females of *An*. *gambiae* sensu lato (s.l.) and *Cx*. *quinquefasciatus* suitable for ovary dissection (live unfed and bloodfed) were processed were to determine parity based on ovary tracheation morphological aspects as described in Detinova [24]. Assessing *An*. *gambiae* s.l. and *Cx*. *quinquefasciatus* parity purposes to determine the physiological age of mosquitoes and the proportion of parous females that are epidemiologically dangerous. Parous females are potentially able to complete successfully the *W*. *bancrofti* stage 1–3 stage lifecycle, and transmit the infective worms (stage 3) to humans. Estimates of mosquito species physiological age an indication of whether a mosquito may survive the extrinsic incubation period of the infecting parasite. The dissected and none-dissected mosquitoes were pooled up to 20 specimens for *An*. *gamabiae* s.l., and up to 30 specimens for *Cx quinquefasciatus* into individual Eppendorf tubes containing silica gel, and stored for subsequent molecular analyses.

### *W*. *bancrofti* DNA detection in mosquitoes

All *An*. *gambiae* s.l. and *Cx*. *quinquefasciatus* mosquitoes that were in good condition (not damaged) were grouped into pools, with a maximum of 20 mosquitoes per pool for *An*. *gambiae* s.l., and 30 per pool for *Cx*. *quinquefasciatus*. In total, 184 pools of *An*. *gambiae* s.l. and 152 pools of *Cx*. *quinquefasciatus* were analyzed by the polymerase chain reaction (PCR) method.

### DNA extraction

Genomic DNA was extracted according to the method of Collins et al. [25] in molecular biology laboratory of the Centre Suisse de Recherches Scientifiques en Côte d’Ivoire (CSRS), Abidjan, Côte d’Ivoire. In brief, whole mosquitoes (*An*. *gambiae* s.l. and *Cx*. *quinquefasciatus*) were soaked in 2% cetyl trimethyl ammonium bromide (CTAB) per pool. The mosquito pools were crushed in 200 μL of CTAB and incubated at 65°C for 5 min. A total of 200 μL of chloroform were added and the resulting mixture was centrifuged for 5 min at 12,000 rpm. The supernatant was pipetted into a new 1.5 mL tube to which 200 μL isopropanol was added; the mixture was centrifuged for 15 min at 12,000 rpm to precipitate the DNA. The supernatant was discarded subsequently, and the DNA pellet formed at the bottom of tubes was purified with 70% ethanol. After a further centrifugation step at 12,000 rpm for 5 min, the ethanol was removed, and the pellet dried on the bench overnight. The extracted DNA was reconstituted in 20 μL DNase-free water (Sigma-Aldrich, United Kingdom) prior to storage at -20°C. Extracted DNA samples were transported using a thermos containing ice-blocks, to the Noguchi Memorial Institute for Medical Research (NMIMR), University of Ghana, Accra, Ghana. These samples were subjected to subsequent molecular analysis.

### Identification of parasite DNA in mosquitoes

Identification of *W*. *bancrofti* DNA in the *An*. *gambiae* s.l. and *Cx*. *quinquefasciatus* mosquitoes was done using polymerase chain reaction (PCR), described in Ramzy et al. [26]. The PCR assay was performed using two oligonucleotides primers, NV-1 (5’- CGT GAT GGC ATC AAA GTA GCG– 3’) and NV-2 (5’–CCC TCA CTT ACC ATA AGA CAA C– 3’). Each amplification reaction was done in a final volume of 10 μL containing 3.2 μL DNase-free water, 5.0 μL of One Taq Quick Load Standard Buffer (2X), 0.4 μL of each primer (0.4 μM) and 1.0 μL DNA template. The PCR was run at an initial denaturation of 94°C for 3 minutes, 35 cycles of denaturation at 94°C for 30 seconds, annealing at 55°C for 1 minute and extension at 68°C for 1 minute, and final extension at 68°C for 5 minutes.

### Data analysis

Data were entered in Microsoft Excel and transferred to STATA 14 (Stata Corp, College Station, Tx, USA) for analysis. The prevalence of *W*. *bancrotfi* among humans was calculated as the percentage of individuals infected with *W*. *bancrofti* among the individuals sampled. The parity rate of the *An*. *gambiae* s.l. and *Cx*. *quinquefasciatus* mosquitoes was the percentage of parous females among females with ovaries dissected. The numbers of parous and nulliparous females were compared using Chi-square. The minimum infection rate of the *An*. *gambiae* s.l. and *Cx*. *quinquefasciatus* mosquitoes was calculated as the percentage of mosquitoes infected with any stage (L1, L2 and/or L3) of the *W*. *bancrofti* parasite. For PCR pooled analyses, the probability that a single mosquito was infected with any stage of *W*. *bancrofti* was calculated using Poolscreen 2.02 software [27], and the maximum likelihood estimates was reported with 95% confidence interval (CI). ArcGIS version 10.2.1 software was used for mapping the study sites.

## Results

### Detection of *W*. *bancrofti* prevalence in people

Table 1 shows the prevalence of *W*. *bancrofti* infection rate measured as CFA by FTS during pre-MDA and post-MDA surveys in the cross-border health districts of Aboisso, Bloléquin, Odienné and Ouangolodougou. Overall, FTS assay indicated that *W*. *bancrofti* CFA rate decreased from 4.60% (n = 739) during the pre-MDA baseline surveys to 0.62% (n = 2,409) in the post-MDA surveys, with a reduction rate of 96.55%. None subjects were found infected with detectable circulating parasite antigen after four rounds of MDA in Ouangolodougou, thus the reduction rate was estimated at 100% (n = 549). Moreover, post-MDA *W*. *bancrofti* CFA rate was lower than 1% in Aboisso (0.19%; n = 526) and Odienné (0.49%; n = 613), with reduction rate of 94.37% and 88.32% compared to pre-MDA baseline outcomes, respectively. However, *W*. *bancrofti* CFA rate was higher than1% with value of 1.53% (n = 721) in Bloléquin. Moreover, the highest number of infected individuals was found in Bloléquin (11/15), followed by Odienné (3/15), and Aboisso (1/15).

**Table 1:** Prevalence of *W*. *bancrofti* infection before and after the mass drug administration in four cross-border health districts of Côte d’Ivoire.

District	Pre-MDA			Post-MDA			
	No. Sampled	CFA rate		No. sampled	CFA rate		Reduction rate
	N	n	%	n	n	%	%
Aboisso	148	5	3.38	526	1	0.19	94.37
Bloléquin	200	11	5.50	721	11	1.53	72.26
Odienné	191	8	4.19	613	3	0.49	88.32
Ouangolodougou	200	10	5.00	549	0	0.00	100
**Total**	**739**	**34**	**4.60**	**2,409**	**15**	**0.62**	**96.55**

n: number, %: percentage, CFA: circulating filarial antigen, MDA: mass drug administration, Pre-MDA corresponds to the period before the first MDA intervention, post-MDA corresponds to the period after the fourth MDA intervention.

After four MDA rounds, a total of 15 individuals was found infected *W*. *bancrofti* in all the study settings (Table 1). The post-MDA data showed that *W*. *bancrofti* Mf were not found in Aboisso, Bloléquin, and Ouangolodougou. However, *W*. *bancrofti* Mf was detected in Odienné, with prevalence of 0.16% (n = 613) and microfilaremia of 32.0 Mf/mL.

**Fig 2:**
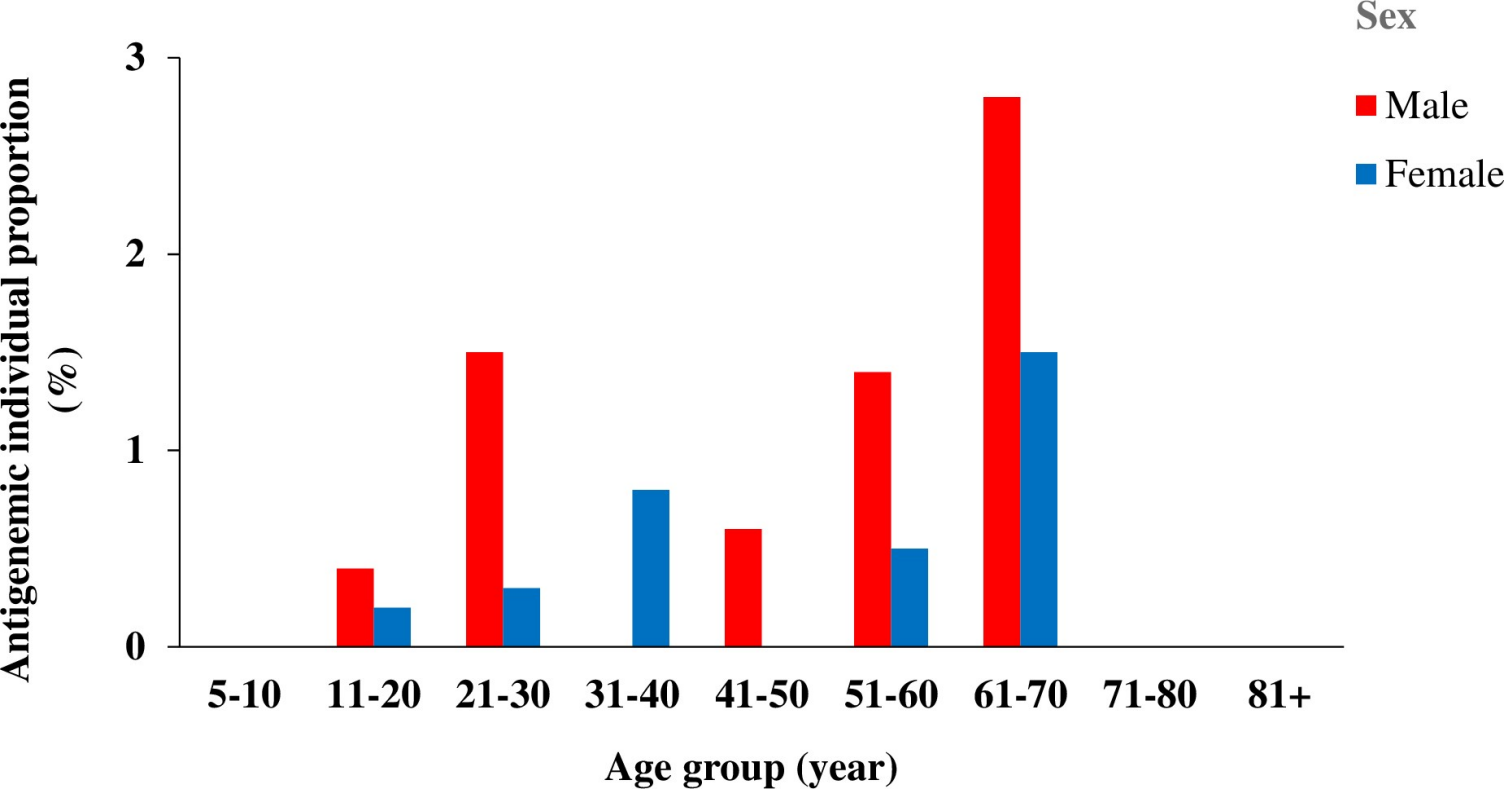
Distribution of individuals infected with *Wuchereria bancrofti* according to the age group and sex in cross-border districts of Côte d’Ivoire after four rounds of MDA. MDA: mass drug administration.

Overall, the proportions of individuals infected with antigenemia remains higher among males (6.7%) than females (3.3%). Fig 2 indicates the proportions of LF antigenemic individuals according to the sex and age group after four rounds of MDA. The proportions of LF antigenemic individuals substantially varied according to the sex and age. Among males, the highest proposition of infected individuals belonged to age group 61–70 years (2.8%), followed by age group 21–30 (1.5%) and age group 51–60 years (1.4%). For females, the majority of infected individuals belonged to age group 61–70 years (1.5%).

### Mosquito species composition

**Fig 3:**
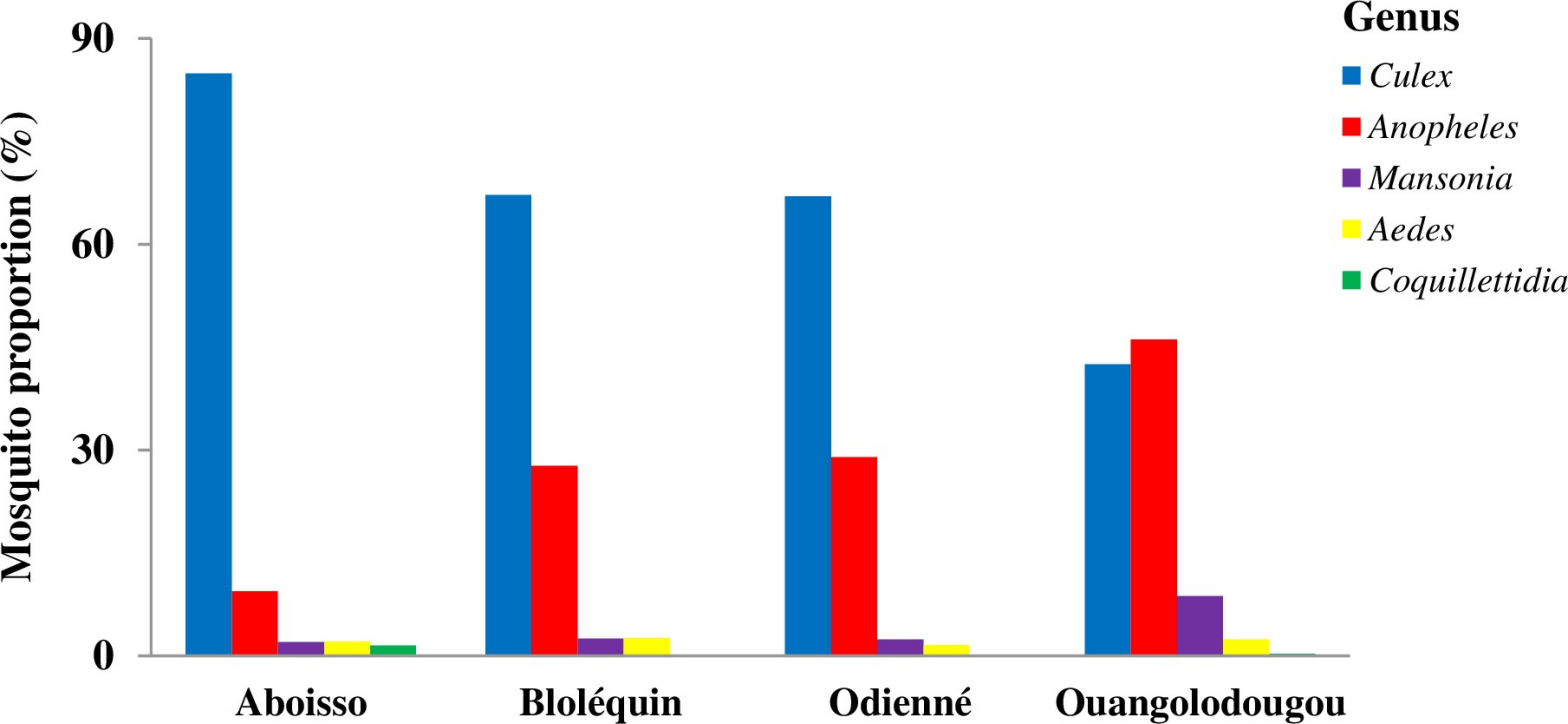
Abundance of mosquito genera collected in cross-border districts of Côte d’Ivoire.

S2 Table indicates the species composition of mosquitoes collected in the cross-border health districts of Aboisso, Bloléquin, Odienné and Ouangolodougou. A total of 15,562 mosquito specimens belonging 26 species grouped into five genera, comprising the potential African vectors of *W*. *bancrofti*, *Culex* (62.15%) and *Anopheles* (30.83%) (Fig 3). Overall, the fauna was dominated by main vectors of *W*. *bancrofti*, namely *An*. *gambiae* s.l. (30.16%; n = 15,562) followed by *Cx*. *quinquefasciatus* (29.24%). *An*. *gambiae* s.l. showed higher abundance in Ouangolodougou (45.64%; n = 5,302), followed by Odienné (28.24%; n = 4,540), Bloléquin (26.25%; n = 2,724), and Aboisso (9.25%; n = 2,996). Conversely, the proposition of *Cx*. *quinquefasciatus* was higher in Aboisso (47.03%; n = 2,996), followed by Bloléquin (29.92%; n = 2,724), Odienné (27.16%; n = 4,540), and Ouangolodougou (20.63; n = 5,302).

### Parity rate of *Anopheles gambiae* s.l. and *Culex quinquefasciatus*

Table 2 presents the parity rates of *An*. *gambiae* s.l. and *Cx*. *quinquefasciatus* species composition of mosquitoes collected in the cross-border health districts of Aboisso, Bloléquin, Odienné, and Ouangolodougou. In general, the parity rates of *An*. *gambiae* s.l. (57.8%; n = 3,094) and *Cx*. *quinquefasciatus* (52.1%; n = 3754) were high. The highest parity rate was observed in *An*. *gambiae* s.l. in Odienné (68.9%; n = 978), followed by Aboisso (63.8%; n = 276), Bloléquin (57.1%; n = 420), and Ouangolodougou (49.2%; n = 1,420). The proportions of parous and nulliparous females of *An*. *gambiae* s.l. were significantly different in Aboisso (χ^2^ = 14.07; df = 1; p <0.001), Bloléquin (χ^2^ = 5.73; df = 1; p = 0.02), and Odienné (χ^2^ = 84.81; df = 1; p <0.001). Similar to *An*. *gambiae* s.l., *Cx*. *quinquefasciatus* displayed higher parity rate in Odienné (57.3%; n = 1,002), followed by followed by Aboisso (56.5%; n = 1,212), Bloléquin (52.3%; n = 692), and Ouangolodougou (39.4%; n = 848). The proportions of parous and nulliparous females of *Cx*. *quinquefasciatus* were statistically different in Aboisso (χ^2^ = 13.76; df = 1; p <0.001), Odienné (χ^2^ = 14.21; df = 1; p <0.001), and Ouangolodougou (χ^2^ = 25.59; df = 1; p <0.001).

**Table 2:** Parity rates of *Anopheles gambiae* s.l. and *Culex quinquefasciatus* mosquitoes in cross-border health districts of Côte d’Ivoire.

District	Mosquito	No. dissected	No. parous	Parity rate		Statistical output		
		n	n	%	95% CI (%)	χ^2^	df	p
Aboisso	*An*. *gambiae* s.l.	276	176	63.8	57.7–69.4	14.07	1	<0.001*
	*Cx*. *quinquefasciatus*	1,212	685	56.5	53.7–59.3	13.76	1	<0.001*
Bloléquin	*An*. *gambiae* s.l.	420	240	57.1	52.2–61.9	5.73	1	0.02*
	*Cx*. *quinquefasciatus*	692	362	52.3	48.5–56.1	0.99	1	0.32
Odienné	*An*. *gambiae* s.l.	978	675	68.9	65.9–71.8	84.81	1	<0.001*
	*Cx*. *quinquefasciatus*	1,002	574	57.3	54.1–60.4	14.21	1	<0.001*
Ouangolodougou	*An*. *gambiae* s.l.	1,420	696	49.0	46.4–51.6	0.37	1	0.54
	*Cx*. *quinquefasciatus*	848	334	39.4	36.1–42.8	25.59	1	<0.001*
Total	*An*. *gambiae* s.l.	3,094	1,787	57.8	56.0–595	49.78	1	<0.001*
	*Cx*. *quinquefasciatus*	3,754	1,955	52.1	50.5–54.0	4.32	1	0.04*

n: number. %: percentage. CI: confidence interval

*: significant difference, χ^2^: chi-square, p: p-value, df: degree of freedom

### Detection of *Wuchereria bancrofti* DNA in *Anopheles gambiae* s.l. and *Culex quinquefasciatus*

Table 3 illustrates the infection rate of *W*. *bancrofti* of *An*. *gambiae* s.l. and *Cx*. *quinquefasciatus* species sampled in the cross-border health districts of Aboisso, Bloléquin, Odienné, and Ouangolodougou. Overall, out of 184 pools of *An*. *gambiae* s.l. and 152 pools of *Cx*. *quinquefasciatus* processed, only 3 and 2 pools were found positive for *W*. *bancrofti* DNA, respectively. None of the analyzed pools of *An*. *gambiae* s.l. and *Cx*. *quinquefasciatus* samples were found infected in Bloléquin, and Ouangolodougou. In contrast, in Aboisso, *An*. *gambiae* s.l. pools (2 positive pools of 11 pools), and *Cx*. *quinquefasciatus* (1 positive pool of 47 pools) were found positive. The pool screening calculation indicated a maximum likelihood estimate (MLE) of infection of 1.0% (95% (confidence interval (CI): 0.12%–3.70%) in *An*. *gambiae* s.l., and 0.07% (95% CI: 0.002%–0.37%) in *Cx*. *quinquefasciatus*. Moreover, in Odienné, *An*. *gambiae* s.l. pools (1 positive pool of 60 pools) and *Cx*. *quinquefasciatus* (1 positive pool of 41 pools) were found infected. MLE of infection was estimated at 0.08% (95% CI: 0.003%–0.43%) for *An*. *gambiae* s.l., and 0.08% (95% CI: 0.002%–0.42%) for *Cx*. *quinquefasciatus*.

**Table 3:** *Wuchereria bancrofti* infection rates in *Anopheles gambiae* and *Culex quinquefasciatus* mosquitoes in four cross-border health districts of Côte d’Ivoire.

District	*Anopheles gambiae*				*Culex quinquefasciatus*			
	No. of pools	PCR positive	Infection rate		No. of pools	PCR positive	Infection rate	
	n	n	%	95% CI	n	n	%	95% CI
Aboisso	11	2	1.00	0.12–3.70	47	1	0.07	0.002–0.37
Bloléquin	30	0	0.00	0.00–0.32	27	0	0.00	0.00–0.24
Odienné	60	1	0.08	0.003–0.43	41	1	0.08	0.002–0.42
Ouangolodougou	83	0	0.00	0.00–0.32	37	0	0.00	0.00–0.17
**Total**	**184**	**3**	**0.0004 **	**0.00008**–**0.001**	**152**	**2**	**0.0003**	**0.00003**–**0.001**

n: number, %: percentage, CI: confidence interval

## Discussion

To our knowledge, our study represents the first study evaluating *W*. *bancrofti* infection indices among human populations and mosquito vectors (i.e., *An*. *gambiae* s.l. and *Cx*. *quinquefasciatus*) in Côte d’Ivoire in connection with MDA interventions to guide LF elimination efforts. Our outcomes showed that *W*. *bancrofti* infection indices were low after the four rounds of MDA interventions in all study areas. MDA might have significantly reduced the infection rate of *W*. *bancrofti* among people living in four cross-border districts, with an overall CFA reduction rate of 96.55%. Moreover, post-MDA *W*. *bancrofti* infection rates among humans were less than the WHO elimination threshold (1%) in three health districts (Aboisso, Odienné and Ouangolodougou), except only for Bloléquin where CFA was above 1%. Moreover, *W*. *bancrofti* prevalence was lower than the WHO thresholds for *An*. *gambiae* (1%), and for *Cx*. *quinquefasciatus* (2%) in the four investigated districts. However, data also appear to show that *An*. *gambiae* s.l. and *Cx*. *quinquefasciatus* were still expected to transmit *W*. *bancrofti* in Aboisso and Odienné. Our study provided valuable findings that may inform further research perspectives and guide future decision regarding MDA cessation and additional efforts to achieve LF EPHP in Côte d’Ivoire. Therefore, the following points are offered for discussion.

First, the pre-MDA surveys carried out among people dwelling in cross-border heath districts of Côte d’Ivoire revealed that *W*. *bancrofti* infection rates were above the threshold (1%), with FTS-positive rate ranged between 3.38% and 5.50%. Although the infection rates in these health districts endemic with LF were quite relatively low, they were all qualified for MDA (CFA rate > 1%) as recommended by the WHO guidelines [4, 7, 17]. It is noteworthy that the LF distribution pattern observed in this study confirms the predictions from a multivariate Bayesian generalized linear spatial model develop to map the distribution of LF across Africa [28]. Similar results have been reported in Gomoa District of Ghana (CFA = 8.7%) by Aboagye-Antwi et al. [29], and two villages of the Democratic Republic of Congo (CFA = 11.8%) by Chesnais et al. [30]. The authors found that occupation-dependent exposure to mosquito bites and no use of bednets are important risk factors for infection with *W*. *bancrofti* [30]. Our study showed that the risk of infection with *W*. *bancrofti* increased with age, and males had higher infection prevalence than females. This suggests that men's body would be more exposed to mosquito bites and infected individuals with adult worms are still present in the communities. Indeed, we found high diversity and high abundance of mosquito species, dominated by *An*. *gambiae* s.l. that is known as a major vector of *W*. *bancrofti* in West Africa [28, 31]. The high abundance of mosquitoes in our study areas might be probably attributable to agricultural activities (e.g., rice growing), low use of bed long-lasting insecticidal nets LLINs distributed by the national malaria programme of Côte d’Ivoire, and local community behaviors.

Second, our data showed a substantial reduction in *W*. *bancrofti* prevalence among people after four MDA rounds compared to the pre-MDA in the cross-border health districts of Côte d’Ivoire. Indeed, a cross-sectional FTS antigen detection survey carried out after the fourth MDA round in these areas revealed a decrease in *W*. *bancrofti* prevalence between 72.26% and 100% compared to the outcomes from the initial baseline pre-MDA survey. Specifically, none subjects were found infected in Ouangolodougou, while *W*. *bancrofti* infection rates were lower than 1% in Aboisso and Odienné. The low *W*. *bancrofti* worm prevalence (< 1%) recorded in Ouangolodougou, Aboisso and Odienné could result from the effects of the MDA interventions. MDA may have significantly reduced the transmission of LF in the study settings [32]. These findings corroborate previous results showing a significant decline in LF Mf prevalence and density in eight of 12 districts in Sierra Leone [33]. Moreover, a community-based study conducted by Pion et al. [34] showed that a 3-year biannual MDA with high therapeutic coverage (more than 80%) of albendazole induced a significant reduction of both *W bancrofti* antigenemia rate (from 17.3% to 4.7%) and microfilaraemia rate (from 5.3% to 0.3%) in the community in all six rounds of MDA in the Republic of the Congo. In the significant reduction observed in our study areas could be due to the relatively low *W*. *bancrofti* prevalence (3.38–5%) recorded during the pre-MDA, and the therapeutic coverage (65–76%) during the MDA compared to the minimum therapeutic coverage threshold (65%). As Mf prevalence fell below the threshold of < 1% in Ouangolodougou, Aboisso and Odienné, MDA interventions should be possibly interrupted, and these districts should be qualified for TAS and molecular xenomonitoring in vectors. However, a TAS may then be implemented to determine the parasite antigen prevalence in 6–7 years old schoolchildren in these cross-border health districts. As *An*. *gambiae* s.l. and *Cx*. *quinquefasciatus* are the primary vectors, if the detected antigenemia in school children is *<*2%, then MDA can be stopped. If the *W*. *bancrofti* infections remain less than 0.25% for *An*. *gambiae* s.l., and 0.3 for *Cx*. *quinquefasciatus*, MDA should stop in the areas. To avoid recrudescence of LF, the TAS should be repeated at years 2–3 and 4–6 after stopping MDA [35], coupled with molecular xenomonitoring of mosquito vectors for LF infection [36].

Third, the *W*. *bancrofti* infection rate (1.53%) was still high and above 1% in the health district of Bloléquin thus revealing ongoing LF transmission. This could thus require the continuation of MDA efforts until the prevalence of worm antigenemia drops below 1%. Similarly, Minetti et al. [37] observed a persistence of LF infection among communities after 10 years of community treatment in rural Ghana. They reported that the prevalence of filarial antigen ranged 0 to 32.4%, and the prevalence of night blood microfilaria (Mf) in five villages was above 1%, while median Mf density was 67 Mf/mL [37]. In the same line, Rao et al. [38] recorded persistent *W*. *Bancrofti* infection in Sri Lanka that has low-level persistence of LF following multiple rounds of MDA in American Samoa. Recent empirical evidence has demonstrated that EPHP does not always lead to elimination of transmission (EOT) [2, 39]. Residual infection remaining after MDA cessation can lead to resurgence and reintroduction [40, 41], with long-term persistence dependent on a range of factors [35], including the presence of *W*. *bancrofti*-infected mosquitoes [2, 39]. Importantly, we detected *W*. *bancrofti* mf in Odienné, even if at low prevalence (0.16%) and low microfilaremia (32.0 Mf/mL). There is a need for an integrated quantitative and qualitative research approach to identify the variations in prevalence, associated risk factors and intervention coverage and use levels within district of Bloléquin. A triple-drug therapy may be recommended to accelerate the EPHP for FL [3, 7, 42, 43]. Moreover, LLINs could be integrated to MDA to accelerate the progress of EPHP and the reduction of LF infection in this health district [2, 39]. Indeed, Jones et al. [44] reported that 12 rounds of MDA complemented with vector control through the use of LLINs resulted in a marked reduction in *W*. *bancrofti* CFA in young school children in Tanzania where mosquitoes were transmitting LF parasites.

Fourth, although our study showed high abundance of potential mosquito vectors throughout, *W*. *bancrofti* DNA was not found in mosquitoes in Bloléquin and Ouangolodougou, but at low prevalence in Aboisso and Odienné (< 2%). LF prevalence was lower than the WHO thresholds of 1% for *An*. *gambiae*, and 2% for *Cx*. *quinquefasciatus* [35, 44, 45] in all study areas. This could support the hypothesis that MDA interventions may have possibly significantly reduced or interrupted the transmission of *W*. *bancrofti* in our study areas [46]. However, the presence of *W*. *bancrofti* in *An*. *gambiae* s.l. and *Cx*. *quinquefasciatus* mosquitoes might raise important issue and merit further discussion. To our knowledge, it is the first time that a study documents their potential implication in the active transmission of *W*. *bancrofti* to humans in Côte d’Ivoire after those conducted by Brengues et al. [19] forty years ago. Indeed, we detected *W*. *bancrofti* DNA in *An*. *gambiae* s.l. and *Cx*. *quinquefasciatus* sampled in the health districts of Aboisso and Odienné. Our study recorded high diversity and abundance of mosquito species, with a strong predominance of *An*. *gambiae* s.l. and *Cx*. *quinquefasciatus* species in the all study areas. The variations in mosquito species diversity and abundance in the investigated areas could be explained by favorable climatic, environmental and ecological conditions, including human activities (e.g., rice farming) and vector control interventions. Although some species belonging to *Anopheles*, *Aedes*, *Culex* and *Mansonia* mosquito genera have been found to carry *W*. *bancrofti* DNA or parasite depending on the geographic location [11], *An*. *gambiae* is known as the main LF vector in West Africa [14, 15], while *Cx*. *quinquefasciatus* mostly transmits the worm in East Africa [46]. Here, we found that *An*. *gambiae* s.l. and *Cx*. *quinquefasciatus* specimens exhibited high parity rates and were infected with *W*. *bancrofti* DNA. Anosike et al. [14] reported *An*. *gambiae* s.l. and *Cx*. *quinquefasciatus* infectivity rates of 6.3% and 6.0% in Nigeria, respectively. Similar findings have been reported in West African countries such as Guinea [47] and Nigeria [48], and in East African countries including Tanzania [49]. A high parity rate is expected to imply that vectors could have sufficient lifespan thus allowing the completion of *W*. *bancrofti* worm life cycle, from stage L1 to L3 before transmitting to humans [2]. The high parity rates recorded may indicate that mosquito vectors may probably survive the extrinsic incubation period of the infecting parasite [50]. PCR-positive mosquitoes provide an indirect indicator of the presence of infected humans and possible ongoing transmission [51]. This would expose the residents of these localities to the risks of both LF and malaria. However, infection rates in the health districts of Aboisso and Odienné were low, and as the molecular-based infection was not stage specific, the infection rate recorded should be interpreted with caution. Besides, it is conceivable that *An*. *gambiae* s.l. and *Cx*. *quinquefasciatus* could still probably support the transmission of *W*. *bancrofti* to people in the cross-border districts of Aboisso and Odienné, and may possibly require complementary vector control actions [52, 53] As *An*. *gambiae* s.l. also transmits malaria *Plasmodium* parasites in Côte d’Ivoire [54], the national malaria control programme should be implicated to facilitate the deployment of malaria vector control (e.g., LLINs) to accelerated the EPHP of LF in these areas [50, 51].

Finally, although our study highlighted a substantial decline in LF prevalence in post-MDA surveys, data are not sufficient to recommend MDA cessation in the health districts of Ouangolodougou, Aboisso and Odienné where the *W*. *bancrofti* infection rate was below LF elimination threshold (1%) established by the WHO. Indeed, the direct statistical comparison between the pre-MDA baseline data collected in 2014 and the present study could not be made due to the unavailability of a detailed methodology and design for the baseline study. Moreover, our analysis was based on the two sentinel sites per district due to financial and logistical limitations. Additional analysis based on the WHO-approved TAS and molecular xenomonitoring in vectors should be implemented at much larger scale in all four districts to confirm our findings. With these methodological limitations, it is advisable that a formal TAS is conducted before the possible cessation of MDA interventions. As Côte d’Ivoire implemented its sixth MDA rounds in April 2019, if the *W*. *bancrofti* infections remain less than 0.25% for *An*. *gambiae* s.l., and 0.3 for *Cx quinquefasciatus* during the TAS phase, MDA should stop in the areas. Above these considerations, our study has significant public health relevance. Indeed, the outcomes provided the important information on *W*. *bancrofti* infection status among both humans and vectors that may update and help with in guiding future decision-making for the national MDA programmes in Côte d’Ivoire. The NPNTDCPC identified 98 of 112 districts as endemic for LF that need MDA to achieve the EPHP. Overall, a maximum of six MDA rounds have been implemented by far, there is a need of scientific and operational focus the district that have received therapeutic treatments. Moreover, the bordering countries have moved to TAS, and a rigorous surveillance of LF transmission in cross-border areas is important to identify and prevent the resurgence of the disease on both sides of the borders [2]. As more West African countries progress towards EPHP [2, 17, 39], it is essential that this process is well-informed, as prematurely halting treatment and surveillance programs could pose a serious threat to global progress [2]. We hope that this study could contribute to understanding drivers of LF elimination and informing MDA long-term policy for a progress towards EPHP in Côte d’Ivoire.

## Conclusion

Overall, in cross-border areas of Côte d’Ivoire, *W*. *bancrofti* infection indices were lower after four rounds of MDA compared to pre-MDA. After MDA, *W*. *bancrofti* CFA in human populations fell below the WHO-established LF elimination threshold (1%) in the health districts of Aboisso, Ouangolodougou and Odienné. However, CFA remained above 1% in Bloléquin. We also recorded a low *W*. *bancrofti* infection (> 2%) in *An*. *gambiae* s.l. and *Cx*. *quinquefasciatus* mosquitoes in the four health districts thus suggesting a low risk of local community exposure to LF transmission. Our data suggested that MDA intervention efforts may have interrupted the transmission of LF in the health districts of Aboisso, Ouangolodougou and Odienné. However, TAS and xenomonitoring surveys in vectors should be recommended prior to eventual cessation of MDA in the three areas. For Bloléquin, MDA efforts should pursue until curving the *W*. *bancrofti* prevalence under 1%. Our results have important ramifications for LF elimination, and MDA implementation policy towards EPHP in the cross-border health districts of Côte d’Ivoire.

## Supporting information

S1 Table Mass drug administration coverage in cross-border health districts of Côte d'Ivoire from 2014 to 2017. (DOCX)

S2 Table Species composition of mosquitoes collected in four cross-border health districts of Côte d’Ivoire. (DOCX)
