# Supplementary material for: Low transmission of Wuchereria bancrofti in cross-border districts of Côte d’Ivoire: A great step towards lymphatic filariasis elimination in West Africa
Source: PLoS One. 2020 Apr 13;15(4):e0231541. doi: 10.1371/journal.pone.0231541 (PMC7153895; doi:10.1371/journal.pone.0231541)
Supplement: S1 Table — (DOCX) [file pone.0231541.s001.docx]

| **S2 Table. Mass drug administration coverage in cross-border health districts of Côte d'Ivoire from 2014 to 2017.** | | | | |
| --- | --- | --- | --- | --- |
| **Heath district** | **2014** | **2015** | **2016** | **2017** |
| Aboisso | 75.4% | 66.8% | 71.5% | 74.6% |
| Bloléquin | 70.3% | 65.6% | 72.2% | 76.6% |
| Odienné | 72.8% | 66.7% | 71.9% | 73.8% |
| Ouangolodougou | 72.0% | 71.1% | 76.4% | 72.8% |
| **Total** | **72,6%** | **67,6%** | **73,0%** | **74,5%** |
| Mass drug adiministartion (MDA) is based on a combination of ivermectin and albendazole | | | | |
