# Supplementary material for: Low transmission of Wuchereria bancrofti in cross-border districts of Côte d’Ivoire: A great step towards lymphatic filariasis elimination in West Africa
Source: PLoS One. 2020 Apr 13;15(4):e0231541. doi: 10.1371/journal.pone.0231541 (PMC7153895; doi:10.1371/journal.pone.0231541)
Supplement: S2 Table — (DOCX) [file pone.0231541.s002.docx]

| **S3 Table. Species composition of mosquitoes collected in four cross-border health districts of Côte d’Ivoire.** | | | | | | | | | | | | | | | | | | | |
| --- | --- | --- | --- | --- | --- | --- | --- | --- | --- | --- | --- | --- | --- | --- | --- | --- | --- | --- | --- |
| **Genus** | **Species** | **Aboisso** | | | | **Bloléquin** | | | | **Odienné** | | | | **Ouangolodougou** | | | | **Total** | |
|  |  | **PSC** | **ETC** | **Total** | | **PSC** | **ETC** | **Total** | | **PSC** | **ETC** | **Total** | | **PSC** | **ETC** | **Total** | |  |  |
|  |  | **n** | **n** | **n** | **%** | **n** | **n** | **n** | **%** | **n** | **n** | **n** | **%** | **n** | **n** | **n** | **%** | **n** | **%** |
| *Culex* | *Cx. quinquefasciatus* | 1,016 | 393 | 1,409 | 47.03 | 569 | 246 | 815 | 29.92 | 721 | 512 | 1,233 | 27.16 | 803 | 291 | 1,094 | 20.63 | 4,551 | 29.24 |
|  | *Cx. nebulosus* | 311 | 223 | 534 | 17.82 | 253 | 275 | 528 | 19.38 | 981 | 430 | 1,411 | 31.08 | 574 | 419 | 993 | 18.73 | 3,466 | 22.27 |
|  | *Cx. cinereus* | 280 | 117 | 397 | 13.25 | 223 | 62 | 285 | 10.46 | 145 | 109 | 254 | 5.59 | 50 | 60 | 110 | 2.07 | 1,046 | 6.72 |
|  | *Cx. decens* | 94 | 46 | 140 | 4.67 | 58 | 37 | 95 | 3.49 | 45 | 18 | 63 | 1.39 | 36 | 4 | 40 | 0.75 | 338 | 2.17 |
|  | *Cx. rima* | 35 | 5 | 40 | 1.34 | 56 | 31 | 87 | 3.19 | 31 | 17 | 48 | 1.06 | 1 | 4 | 5 | 0.09 | 180 | 1.16 |
|  | *Cx. tigripes* | 3 | 0 | 3 | 0.10 | 4 | 1 | 5 | 0.18 | 6 | 7 | 13 | 0.29 | 9 | 0 | 9 | 0.17 | 30 | 0.19 |
|  | *Cx. annulioris* | 12 | 3 | 15 | 0.50 | 2 | 1 | 3 | 0.11 | 2 | 4 | 6 | 0.13 | 1 | 1 | 2 | 0.04 | 26 | 0.17 |
|  | *Cx. poicilipes* | 7 | 0 | 7 | 0.23 | 1 | 6 | 7 | 0.26 | 5 | 0 | 5 | 0.11 | 2 | 0 | 2 | 0.04 | 21 | 0.13 |
|  | *Cx. perfuscus* | 0 | 0 | 0 | 0.00 | 3 | 2 | 5 | 0.18 | 9 | 0 | 9 | 0.20 | 0 | 0 | 0 | 0.00 | 14 | 0.09 |
|  | **Total** | **1,758** | **787** | **2,545** | **84.95** | **1,169** | **661** | **1,830** | **67.18** | **1,945** | **1,097** | **3,042** | **67.00** | **1,476** | **779** | **2,255** | **42.53** | **9,672** | **62.15** |
| *Anopheles* | *An. gambiae* s.l. | 126 | 151 | 277 | 9.25 | 479 | 236 | 715 | 26.25 | 741 | 541 | 1,282 | 28.24 | 1,142 | 1,278 | 2,420 | 45.64 | 4,694 | 30.16 |
|  | *An. funestus* | 0 | 2 | 2 | 0.07 | 20 | 12 | 32 | 1.17 | 10 | 13 | 23 | 0.51 | 5 | 6 | 11 | 0.21 | 68 | 0.44 |
|  | *An. nili* | 0 | 2 | 2 | 0.07 | 1 | 0 | 1 | 0.04 | 3 | 1 | 4 | 0.09 | 0 | 0 | 0 | 0.00 | 7 | 0.04 |
|  | *An. coustani* | 0 | 0 | 0 | 0.00 | 0 | 0 | 0 | 0.00 | 0 | 0 | 0 | 0.00 | 6 | 1 | 7 | 0.13 | 7 | 0.04 |
|  | *An. pharoensis* | 0 | 0 | 0 | 0.00 | 1 | 0 | 1 | 0.04 | 1 | 2 | 3 | 0.07 | 2 | 0 | 2 | 0.04 | 6 | 0.04 |
|  | *An. cinctus* | 0 | 0 | 0 | 0.00 | 0 | 0 | 0 | 0.00 | 3 | 0 | 3 | 0.07 | 3 | 0 | 3 | 0.06 | 6 | 0.04 |
|  | *An. domicolus* | 0 | 0 | 0 | 0.00 | 1 | 0 | 1 | 0.04 | 4 | 0 | 4 | 0.09 | 0 | 0 | 0 | 0.00 | 5 | 0.03 |
|  | *An. paludis* | 0 | 0 | 0 | 0.00 | 4 | 0 | 4 | 0.15 | 0 | 0 | 0 | 0.00 | 0 | 0 | 0 | 0.00 | 4 | 0.03 |
|  | **Total** | **126** | **155** | **281** | **9.38** | **506** | **248** | **754** | **27.68** | **762** | **557** | **1,319** | **29.05** | **1,158** | **1,285** | **2,443** | **46.08** | **4,797** | **30.83** |
| *Mansonia* | *Ma. Africana* | 12 | 24 | 36 | 1.20 | 19 | 22 | 41 | 1.51 | 66 | 30 | 96 | 2.11 | 120 | 131 | 251 | 4.73 | 424 | 2.72 |
|  | *Ma. uniformis* | 15 | 10 | 25 | 0.83 | 12 | 16 | 28 | 1.03 | 9 | 2 | 11 | 0.24 | 45 | 163 | 208 | 3.92 | 272 | 1.75 |
|  | **Total** | **27** | **34** | **61** | **2.04** | **31** | **38** | **69** | **2.53** | **75** | **32** | **107** | **2.36** | **165** | **294** | **459** | **8.66** | **696** | **4.47** |
| *Aedes* | *Ae. aegypti* | 48 | 11 | 59 | 1.97 | 38 | 24 | 62 | 2.28 | 41 | 25 | 66 | 1.45 | 83 | 25 | 108 | 2.04 | 295 | 1.90 |
|  | *Ae. dendrophilus* | 0 | 1 | 1 | 0.03 | 0 | 1 | 1 | 0.04 | 1 | 1 | 2 | 0.04 | 3 | 11 | 14 | 0.26 | 18 | 0.12 |
|  | *Ae. africanus* | 0 | 2 | 2 | 0.07 | 0 | 0 | 0 | 0.00 | 0 | 0 | 0 | 0.00 | 4 | 1 | 5 | 0.09 | 7 | 0.04 |
|  | *Ae. vittatus* | 0 | 0 | 0 | 0.00 | 3 | 0 | 3 | 0.11 | 4 | 0 | 4 | 0.09 | 0 | 0 | 0 | 0.00 | 7 | 0.04 |
|  | *Ae. longipalpis* | 0 | 0 | 0 | 0.00 | 4 | 0 | 4 | 0.15 | 0 | 0 | 0 | 0.00 | 1 | 0 | 1 | 0.02 | 5 | 0.03 |
|  | *Ae. opok* | 1 | 0 | 1 | 0.03 | 0 | 1 | 1 | 0.04 | 0 | 0 | 0 | 0.00 | 0 | 0 | 0 | 0.00 | 2 | 0.01 |
|  | ***Aedes*** | **49** | **14** | **63** | **2.10** | **45** | **26** | **71** | **2.61** | **46** | **26** | **72** | **1.59** | **91** | **37** | **128** | **2.41** | **334** | **2.15** |
| *Coquelitidia* | *Cq. Cristata* | 46 | 0 | 46 | 1.54 | 0 | 0 | 0 | 0.00 | 0 | 0 | 0 | 0.00 | 9 | 8 | 17 | 0.32 | 63 | 0.40 |
|  | **Total** | **46** | **0** | **46** | **1.54** | **0** | **0** | **0** | **0.00** | **0** | **0** | **0** | **0.00** | **9** | **8** | **17** | **0.32** | 63 | 0.40 |
| **Total** | **Abundance** | **2,006** | **990** | **2,996** | **100** | **1,751** | **973** | **2,724** | **100** | **2,828** | **1,712** | **4,540** | **100** | **2,899** | **2,403** | **5,302** | **100** | **15,562** | **100** |
|  | **No. of species** | **14** | **14** | **18** | **na** | **20** | **16** | **22** | **na** | **20** | **15** | **20** | **na** | **20** | **15** | **20** | **na** | **26** | **na** |
| n: number, %: percentage, ETC: Exit trap collection, PSC: Pyrethrum knock-down spray collection, na: not applicable | | | | | | | | | | | | | | | | | | | |
